# Mitochondrial Ferritin Protects Hydrogen Peroxide-Induced Neuronal Cell Damage

**DOI:** 10.14336/AD.2016.1108

**Published:** 2017-07-21

**Authors:** Guofen Gao, Nan Zhang, Yue-Qi Wang, Qiong Wu, Peng Yu, Zhen-Hua Shi, Xiang-Lin Duan, Bao-Lu Zhao, Wen-Shuang Wu, Yan-Zhong Chang

**Affiliations:** ^1^Laboratory of Molecular Iron Metabolism, College of Life Science, Hebei Normal University, Shijiazhuang, Hebei, 050024, China; ^2^The 3rd Hospital of Hebei Medical University, Shijiazhuang, Hebei, 050017, China

**Keywords:** mitochondrial ferritin, hydrogen peroxide, iron, oxidative stress, labile iron pool

## Abstract

Oxidative stress and iron accumulation are tightly associated with neurodegenerative diseases. Mitochondrial ferritin (FtMt) is identified as an iron-storage protein located in the mitochondria, and its role in regulation of iron hemeostasis in neurodegenerative diseases has been reported. However, the role of FtMt in hydrogen peroxide (H_2_O_2_)-induced oxidative stress and iron accumulation in neuronal cells has not been studied. Here, we overexpressed FtMt in neuroblastoma SH-SY5Y cells and induced oxidative stress by treating with extracellular H_2_O_2_. We found that overexpression of FtMt significantly prevented cell death induced by H_2_O_2_, particularly the apoptosis-dependent cell death. The protective effects involved inhibiting the generation of cellular reactive oxygen species, sustaining mitochondrial membrane potential, maintaining the level of anti-apoptotic protein Bcl-2, and inhibiting the activation of pro-apoptotic protein caspase 3. We further explored the mechanism of these protective effects and found that FtMt expression markedly altered iron homeostasis of the H_2_O_2_ treated cells as compared to that of controls. The FtMt overexpression significantly reduced cellular labile iron pool (LIP) and protected H_2_O_2_-induced elevation on LIP. While in H_2_O_2_ treated SH-SY5Y cells, the increased iron uptake and reduced iron release, in correlation with levels of DMT1(-IRE) and ferroportin 1, resulted in heavy iron accumulation, the FtMt overexpressing cells didn’t show any significant changes in levels of iron transport proteins and in the level of LIP. These results implicate a neuroprotective role of FtMt on H_2_O_2_-induced oxidative stress, which may provide insights into the treatment of iron accumulation associated neurodegenerative diseases.

Iron is an essential component for activities of numerous proteins and enzymes that are critical for cell respiration, proliferation and signal transduction [1, 2]. Iron metabolism in the body is strictly controlled, and disruption of iron homeostasis would cause severe diseases. Recently, neurodegenerative disorders that associated with iron accumulation in neurons, such as Parkinson’s and Alzheimer’s diseases, have attracted more and more attention [3-5]. Excess iron is highly toxic because of its ability to form highly reactive hydroxyl radicals in the presence of hydrogen peroxide (H_2_O_2_) [6]. The production of these reactive oxygen species (ROS) in neurons can damage cellular macromolecules including proteins, lipids and DNA, and finally the triggered oxidative stress will induce apoptosis of the neurons [7, 8].

Ferritin is a ubiquitous iron-storage protein in the cytosol that forms a spherical shell by 24 subunits and accommodates up to 4500 iron atoms [9, 10]. Most intracellular iron is stored in the cytosol by binding to ferritin. In mammalian cells, two ferritin subtypes have been found, H-ferritin and L-ferritin. The former subtype has ferroxidase activity essential for the storage of free iron in ferritin, while the latter has a nucleation site that is involved in iron-core formation [11, 12]. It has been reported that ferritins exert cellular protective roles against iron-mediated free radical damage induced by a variety of sources [10, 13]. In neuronal cells, elevated ferritin expression has been shown to protect the MPTP-induced experimental PD models well [14]. Therefore, it appears that neuronal cell survival is also dependent on the cellular level of ferritin.

Mitochondrial ferritin (FtMt) is a ferritin type protein targeted to mitochondria, and has been characterized structurally and functionally analogous to the well-characterized cytosolic H-ferritin [15]. FtMt has been shown to modulate cellular iron metabolism dramatically [16-19]. Previous studies suggested that overexpression of FtMt caused redistribution of iron from cytosol to mitochondria [16, 19], thus high levels of FtMt resulted in an iron deficient phenotype in cytosol [20]. The expression of FtMt is restricted to mitochondria of cells of testes, the central nervous system, and some other high oxygen-consumption tissues [21], indicating that the major role of FtMt may be protecting mitochondria from iron-dependent oxidative damage in cells characterized by high metabolic activity and oxygen consumption [22]. Our previous studies have found that FtMt overexpression protected 6-hydroxydopamine-induced dopaminergic cell damage, potentially playing an important neuroprotective role in Parkinson’s Disease [23]. Studies by Campanella *et al.* revealed a protective role of FtMt in Friedreich ataxia, a disease characterized by mitochondrial iron overload and oxidative damage [24, 25]. FtMt expression also inhibited tumor growth due to cytosolic iron deprivation [26]. The protective role of FtMt against oxidative stress in other disease models has also been suggested [22, 27-30]. These studies demonstrated that FtMt is not only involved in storing cellular iron, but may also play a role in protecting mitochondria from iron-dependent oxidative damage [22-30].

In this study, we aimed to investigate the role that FtMt plays against the oxidative stress to mitochondria induced by H_2_O_2_. A recent study by Dev *et al.* revealed the effect of H_2_O_2_ treatment on LIP level and cellular iron-uptake, -storage and -release proteins in the neuroblastoma cell line SH-SY5Y [31]. They found that iron heavily accumulated in SH-SY5Y cells after H_2_O_2_ treatment, and iron-release protein FPN1 significantly decreased, whereas iron-uptake protein didn’t change much [31]. Interestingly, they also found the expression of iron-storage protein H-ferritin was decreased, which was not in accordance with the regulation by the iron-regulatory protein (IRP) [32]. However, the functions of the iron-storage protein in mitochondria, FtMt, in H_2_O_2_ induced oxidative stress in neuronal cells have not been studied. We hypothesized that FtMt may play a neuroprotective role in H_2_O_2_ induced cell stress. Thus, we overexpressed FtMt gene in the neuroblastoma SH-SY5Y cells to see if an increase of FtMt expression can sequester more free iron and counter the H_2_O_2_-induced iron accumulation and cell damage. We further investigated its effects on iron metabolism and the mechanisms of neuroprotection in H_2_O_2_-induced apoptosis. This study would be useful for understanding the roles of FtMt in neurodegenerative diseases. It may provide insight into discovering new therapeutic methods for treatment of iron overload-related neurodegenerative disorders.

## MATERIALS AND METHODS

### Cell lines and H_2_O_2_ treatment

The stable FtMt-expressing SH-SY5Y cell line (FtMt-SY5Y) and pcDNA3.1(-) empty vector transfected cell line (vector-SY5Y) were generated as described previously [23]. Briefly, the amplified mouse FtMt cDNA, with a C-terminal hemagglutinin (HA) epitope sequence, was cloned into pcDNA3.1(-) vector to generate construct FtMt-pcDNA3.1(-)[20]. The plasmids of FtMt-pcDNA3.1(-) and empty vector were transfected into SH-SY5Y cells, and stable transfectants were selected [23]. The expression of mouse FtMt protein was confirmed with western blotting by using anti-HA antibody [23]. The endogenous expression of human FtMt in SH-SY5Y cells was very low as compared to the levels of overexpressed mouse FtMt as measured by RT-PCR [23]. The cell viability and apoptotic ratio of FtMt-SY5Y cells had no difference compared to the wild-type (WT) SH-SY5Y cells [23], but the growth of FtMt-SY5Y cells was significantly slower [26].

The WT SH-SY5Y cells, FtMt-SY5Y cells and vector-SY5Y cells were maintained in Dulbecco modified Eagle medium supplemented with 10% fatal bovine serum, 100 U/ml penicillin and 100 U/ml streptomycin. After the cells reaching ~80% confluency, H_2_O_2_ were added to a final concentration of 100 μM (or as described in each specific experiment), and cells were then incubated at 37? for 24 h prior to analysis.

### Antibodies and Chemicals

Rabbit anti-human β-actin antibody, rabbit anti-rat DMT1(+IRE) antibody, rabbit anti-rat DMT1(-IRE) antibody, anti-mouse FPN1 antibody and anti-rat TfR1 antibody were purchased from ADI (San Antonio, TX, USA); anti-human H-ferritin and L-ferritin antibodies were purchased from Abcam (Cambridge, MA, USA); anti-human caspase 3 antibody, and anti-mouse Bax and Bcl-2 antibody were purchased from Santa Cruz Biotechnology (Santa Cruz, CA, USA); rabbit anti-mouse FtMt polyclonal antibody [21] and mouse anti-human FtMt monoclonal antibody [33] were gifts from Prof. Sonia Levi. The salicylaldehyde isonicotinoyl hydrazone (SIH) was synthesized as described by Ponka *et al.* [34]. Unless otherwise stated, all chemicals were purchased from Sigma Chemical Co. (St. Louis, MO, USA).

### Western blotting

After H_2_O_2_ treatment (100 μM, 24 h), cells were homogenized and lysed with RIPA buffer and protein content was determined by the Bradford assay. Aliquots of cell lysate containing approximately 30 μg of protein were immediately mixed with loading buffer and boiled for 10 minutes. Equal amounts of protein for different cells were loaded, resolved by sodium dodecyl sulfate-polyacrylamide gel electrophoresis (SDS-PAGE) and transferred to a PVDF membrane. The blots were blocked by incubating with 5% nonfat milk in PBS containing 0.1% Tween 20 (PBS-T) for 1 h, and hybridized with primary antibodies. After washing 3 times for 15 minutes each with PBS-T, the blots were incubated for 1 h with peroxidase-coupled secondary antibodies, and detected with the ECL plus Western Blotting Detection Reagents (Pierce Biotechnology, Rockford IL). Six independent experiments were performed for each treatment.

### Cell viability assay

Cell viability was assessed by vital dye reduction with 3-(4, 5-dimethylthiazol-2-yl)-2, 5-diphenyl-tetrazolium bromide (MTT) [35]. Briefly, SH-SY5Y cells were seeded in a 96-well plate at a density of 2×10^4^ cells/well. After attachment, H_2_O_2_ was added to cell culture in DMEM without serum at final concentrations of 0, 20, 40, 60, 80, 100, 120, 140 μmol/L, and incubated for the subsequent 24 h at 37?. H_2_O_2_ treated or untreated cells were incubated in MTT (0.5 mg/mL) for another 3 h, and then cells were lysed with DMSO, and the absorbance at 595 nm was measured with a Bio-Rad model 3550 microplate reader (Richmond, CA). All samples were measured in six independent experiments.

### Detection of cell apoptosis

After H_2_O_2_ treatment (100 μM, 24 h), cell apoptosis was detected by using annexin V/PI assay by flow cytometry as described previously [23].

### Measurement of the labile iron pool (LIP) level

The LIP level was determined by the quenching of calcein fluorescence as described previously [36, 37]. After treatment with or without 100 μM H_2_O_2_ for 24 h, the cells were harvested, washed, and resuspended in PBS (pH 7.4) buffer containing calcein-AM (0.25 μM final concentration), and incubated for 30 min at 37?. The excess calcein-AM on the cell surface was washed out 3 times with PBS. The fluorescence intensity of calcein-AM was quantified by a fluorescence spectrophotometer (Hitachi F-4500), at an excitation wavelength of 488 nm and an emission wavelength of 525 nm. When the baseline was stable, SIH (100 μM final concentration) was added, and the increase in fluorescence intensity reflected the levels of calcein-bound iron. Three independent experiments were performed for each treatment.

### Detection of intracellular oxidative stress-ROS assay

Intracellular ROS were examined using 2′,7′-dichlorofluorescein diacetate (DCF-DA) as described before [38]. After treatment with or without 100 μM H_2_O_2_ for 24 h, cells were harvested, washed, and resuspended in PBS containing DCF-DA (10 μM final concentration), and incubated for 30 min at 37?. Cells were washed twice with PBS, and the fluorescence signals were measured by a fluorescence spectrophotometer with 488 nm excitation and 525 nm emission wavelengths. Three independent experiments were performed for each treatment. The data were expressed as a percentage of the fluorescence relative to the fluorescence of the untreated WT control cells.

### Detection of mitochondrial membrane potential (MMP)

Changes in the MMP with or without H_2_O_2_ treatment (100 μM, 24 h) of SH-SY5Y cells were determined by measuring the retention of rhodamine 123 using flow cytometry [39]. The uptake of rhodamine123 into mitochondria is an indicator of the MMP. Cells were incubated with rhodamine123 at a final concentration of 5 μM for 30 min at 37?. After washing twice with PBS, fluorescence was recorded at 488 nm excitation and 525 nm emission wavelengths. Three independent experiments were performed for each treatment.

### Measurement of iron uptake and release

^55^Fe (^55^FeCl_3_, Perkin-Elmer Life Sciences Company, Wellesley City, MA) solution was prepared by mixing ^55^FeCl_3_ with FeSO_4_ in a molar ratio of 1:10 followed by a 50-fold molar excess of 2-mercaptoethanol and 0.27 M sucrose (pH 6.5) as described previously [40, 41]. The H_2_O_2_ treated (100 μM, 24 h) or untreated cells (about 1×10^6^ cells) were washed three times with PBS buffer, and added with ^55^FeCl_3_ solution (1 mM) for 30 min at 37? and then washed three times with PBS. The cells were homogenized in buffer containing 1% sodium dodecyl sulphate (SDS), and aliquots of the total cell extract were assayed for released radioactivity with Liquid Scintillation Analyzer (Beckman) and protein concentrations by Lowry method. Three independent experiments were performed for each treatment.

For measurement of iron release, the cells were incubated with ^55^FeCl_3_ solution for 30 min and then washed three times with PBS. The cells were then incubated with 1 ml PBS at 37? for 30 min. The supernatant was collected. The cells were homogenized after washing three times with PBS. Both supernatant and cell extract were assayed for radioactivity. Percentage (%) of ^55^Fe release = (cpm in supernatant) / (cpm in supernatant + cpm in cells) × 100%.

**Figure 1.**
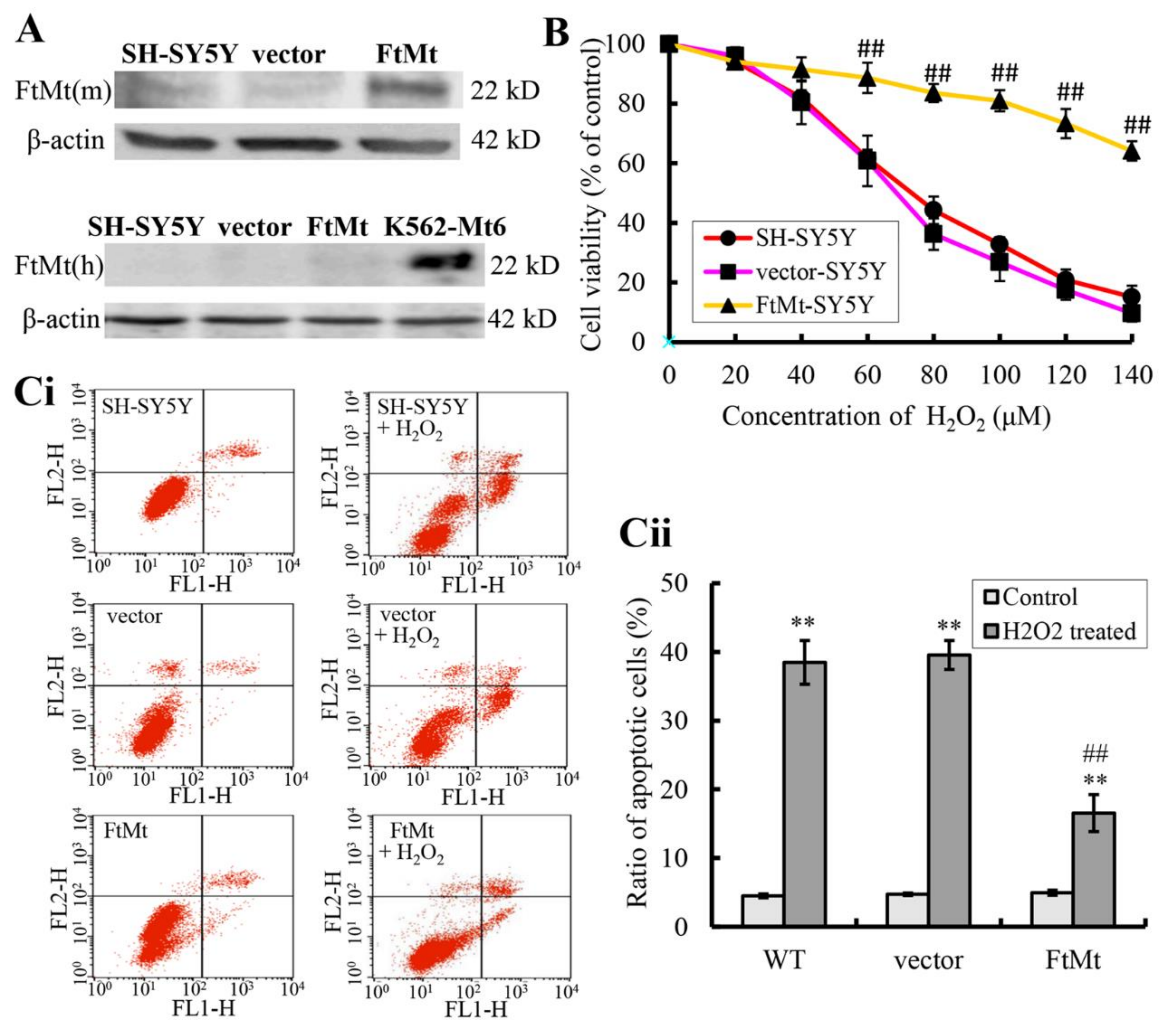
Effects of FtMt on cell viability and apoptosis ratios of cells treated with H_2_O_2_ (**A**) The expressions of exogenous mouse FtMt (top panel) and endogenous human FtMt (bottom panel) in FtMt overexpressed transfectants (FtMt) were examined by western blot using anti-mouse and anti-human FtMt antibodies, respectively. K562-Mt6 is a human FtMt overexpressing cell line (a gift from Prof. Sonia Levi), which is used as a positive control. (**B**) The wild-type (WT) SH-SY5Y cells, empty vector transfectants (vector), and FtMt overexpressed transfectants (FtMt) were treated with increasing concentrations (0, 20, 40, 60, 80, 100, 120, 140 μM) of H_2_O_2_ for 24 h. Cell viability was measured with the MTT assay. Data were presented as percentage of the cell viability compared with untreated WT control cells ± SD, n=6 (##, *p* < 0.01 vs. the H_2_O_2_-treated vector control cells). (**C**) The relative apoptotic ratios of control and FtMt-SY5Y cells with or without H_2_O_2_ treatment (100 μM, 24 h) were determined by flow-cytometry (Ci and Cii). Data were presented as means ± SD; n=3 (**, *p* < 0.01 vs. the untreated cells of same genotype; ##, *p* < 0.01 vs. the H_2_O_2_-treated vector controls).

### Statistical analysis

All statistical analyses were completed using SPSS 21.0 software. Results are presented as means ± SD. The statistical analyses of group differences were assessed by a two-way analysis of variance (ANOVA). *P*-values of <0.05 were considered to be statistically significant; *P* <0.01 was considered to be remarkably significant.

## RESULTS

### FtMt overexpression rescued neuronal cell death induced by H_2_O_2_ treatment

Previous studies have indicated that FtMt plays an important role in protecting cells from iron-dependent oxidative damage [23, 25, 27, 30], particularly in neurodegenerative diseases [42, 43]. In order to investigate the role of FtMt in hydrogen peroxide (H_2_O_2_)-induced neuronal cell damage, a stable FtMt-overexpressing neuroblasma cell line, FtMt-SY5Y, generated previously was used [23]. Wild-type SH-SY5Y cells and pcDNA3.1(-) empty plasmid transfected cells (vector-SY5Y) were used as controls. The expression of FtMt in FtMt-SY5Y cells was confirmed (Fig. 1A). The level of overexpressed mouse FtMt was much higher than that of the endogenous human FtMt. The cell viability of the three cell lines was determined after H_2_O_2_ treatment for 24 h. Results showed that H_2_O_2_ reduced cell viability in all three cell lines in a concentration-dependent manner (Fig. 1B). However, after H_2_O_2_ treatment, at concentrations of 60, 80, 100, 120 and 140 μM, cell viability in FtMt-SY5Y cells was significantly higher than that of the vector-SY5Y cells (*P* < 0.01, Fig. 1B). With 100 μM H_2_O_2_ treatment, the viability of SH-SY5Y and vector-SY5Y cells were decreased to ~40%, whereas the viability of FtMt-SY5Y cells only had a ~11% decrease. 100 μM of H_2_O_2_ treatment was then used for the following assays. These results indicated that H_2_O_2_ induced cell damage and that overexpression of FtMt in SH-SY5Y cells improved cell viability.

**Figure 2.**
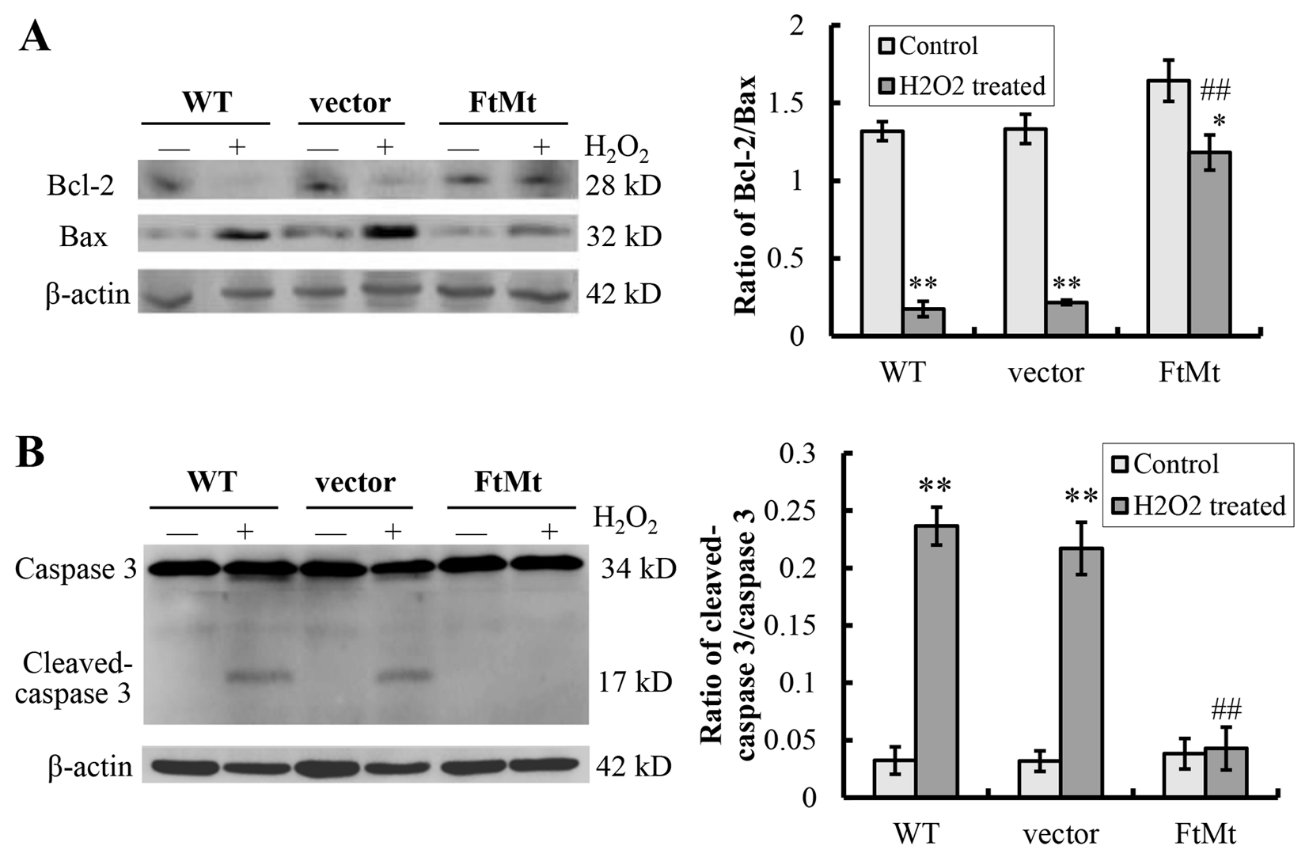
FtMt protected cells from apoptosis by maintaining Bcl-2/Bax ratio and inhibiting activation of caspase 3 (**A**) The protein expression levels of Bcl-2 and Bax in H_2_O_2_ treated (100 μM, 24 h) or untreated cells were determined by western-blot (right panel), and ratios of Bcl-2/Bax were calculated and presented as mean ± SD (left panel); n=6 (*, *P* < 0.05 and **, *P* < 0.01 vs. the untreated cells of same genotype; ##, *p* < 0.01 vs. the H_2_O_2_-treated vector controls). (**B**) Expression levels of caspase 3 and cleaved-caspase 3 were shown (right panel). Ratios of cleaved-caspase 3/caspase 3 were calculated and presented as mean ± SD (left panel); n=6 (**, *P* < 0.01 vs. the untreated cells of same genotype; ##, *p* <0 .01 vs. the H_2_O_2_-treated vector controls).

**Figure 3.**
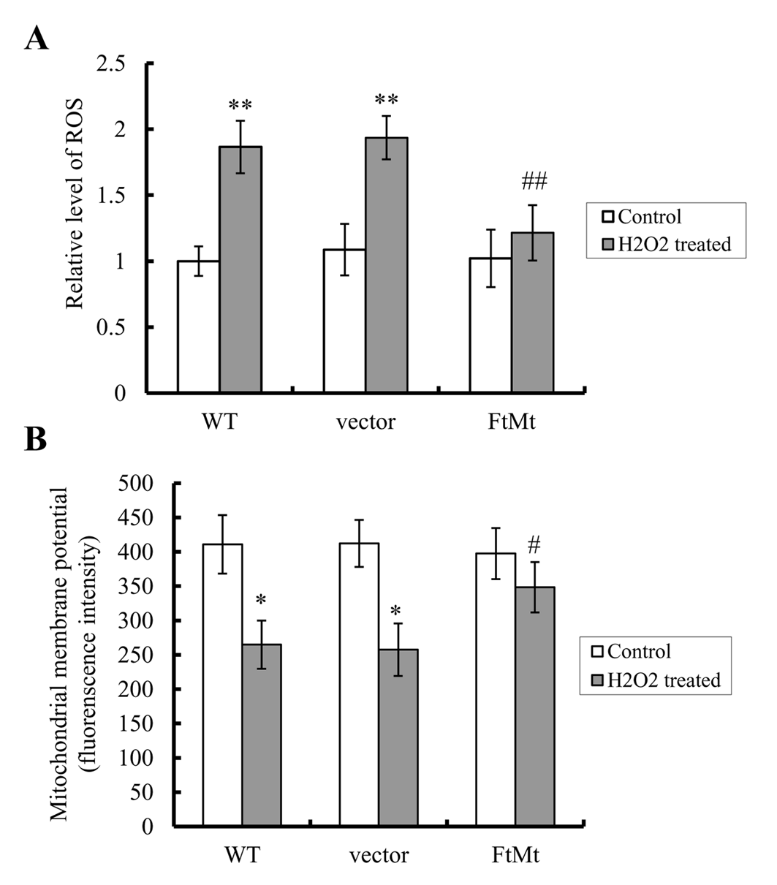
FtMt attenuated H_2_O_2_-induced increases in ROS level and H_2_O_2_-induced decreases in MMP (**A**) Cells treated with or without 100 μM H_2_O_2_ for 24 h were detected for ROS production by 2,7-dichlorofluorescein diacetate (DCF-DA) fluorescence. The relative fluorescence level for each group against that of the WT untreated control is expressed as mean ± SD, n=3 (**, *P* < 0.01 vs. the untreated cells of same genotype; ##, *p* < 0.01 vs. the H_2_O_2_-treated vector controls). (**B**) The MMP of H_2_O_2_ treated (100 μM, 24 h) or untreated cells was detected by the fluorescence of rhodamine 123. Data are presented as means of fluorescence intensity ± SD, n=3 (*, *P* < 0.05 vs. the untreated cells of same genotype; #, *p* <0.05 vs. the H_2_O_2_-treated vector controls).

To explore if the rescue effect of FtMt was resulted from increased resistance of FtMt-SY5Y cells against apoptosis induced by H_2_O_2_, the apoptotic rates in vector-SY5Y and FtMt-SY5Y cells were quantified by flow cytometry after annexin V and PI staining. After treated with 100 μM H_2_O_2_ for 24 h, the ratio of apoptotic vector-SY5Y cells increased from 4.7% to 39.5% (*P* < 0.01, Fig. 1Cii, indicated by **); the apoptotic ratio of FtMt-SY5Y cells also increased (from 4.9% to 16.5%, *P* < 0.01). However, when comparing the increases between FtMt overexpressing cells and vector control cells, the FtMt overexpressing cells had significantly lower number of apoptotic cells (*P* < 0.01, Fig. 1C, indicated by ##). The results inferred that FtMt has a significant anti-apoptotic role in H_2_O_2_-induced cell damage.

### FtMt maintained Bcl-2/Bax ratio and inhibited the activation of caspase 3

Bcl-2 and Bax are anti-apoptotic and pro-apoptotic proteins, and the Bcl-2/Bax ratio has been widely used to monitor apoptosis [44]. To further confirm if the rescue effect of FtMt on cell death was resulted from decreasing cell apoptosis, the ratios of Bcl-2/Bax for vector-SY5Y and FtMt-SY5Y cells with/without H_2_O_2_ treatment were quantified by western blotting. In vector-SY5Y cells, dramatic decreases in Bcl-2 levels and increases in Bax levels were observed after H_2_O_2_ treatment as compared to that of the untreated control group, causing obviously reduced Bcl-2/Bax ratios (*P* < 0.01, Fig. 2A). However, the FtMt over-expressed FtMt-SY5Y cells maintained the levels of both Bcl-2 and Bax, showing a relatively small change in the Bcl-2/Bax ratio (*P* < 0.05, Fig. 2A). H_2_O_2_ treatment also caused caspase 3 activation in SH-SY5Y cells and vector-SY5Y cells, as shown by the increased cleaved-caspase 3 levels (Fig. 2B), whereas the activation wasn’t observed in the FtMt-SY5Y cells. These findings indicate that FtMt significantly maintains the normal level of Bcl-2/Bax and prevents the activation of caspase 3 to protect the cells against H_2_O_2_-induced apoptosis, thereby protecting cells from death.

### FtMt attenuated H_2_O_2_-induced increases in ROS level and H_2_O_2_-induced decreases in mitochondrial membrane potential (MMP)

Evidences have strongly suggested that apoptosis is closely linked to production of damaging ROS during electron transport [7, 45], therefore we determined the effect of FtMt expression on the ROS level, by measuring the ROS-dependent fluorescence of DCF-DA using flow cytometry [38]. The relative ROS levels of different cells with/without H_2_O_2_ treatment and their differences are shown in Fig. 3A. The H_2_O_2_ treatments led to significant increases in intracellular ROS production in both SH-SY5Y and vector-SY5Y cells as compared to the untreated controls (*P* < 0.01). In contrast, the ROS level of FtMt-SY5Y cells only increased slightly by H_2_O_2_ treatment as compared to the untreated control. This indicates that although the overexpression of FtMt doesn’t alter the intracellular ROS levels in SH-SY5Y cells under normal conditions, it can significantly attenuate the H_2_O_2_-induced increase in ROS level, which therefore protects the SH-SY5Y cells from oxidative damage.

**Figure 4.**
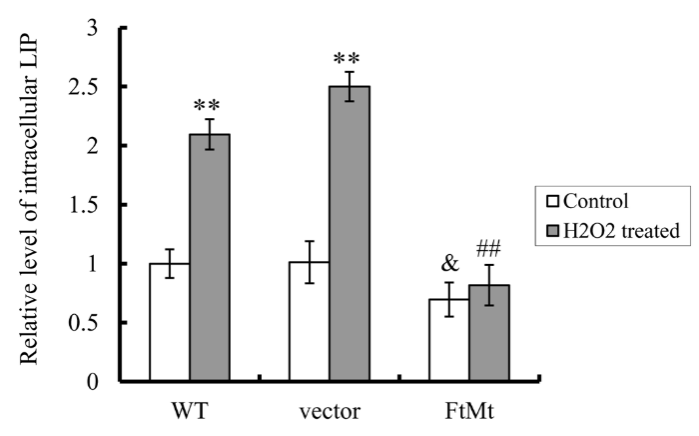
FtMt overexpression significantly decreased cellular LIP levels in both H_2_O_2_ treated (100 μM, 24 h) and untreated cells LIP levels were determined by the quenching of calcein fluorescence method using fluorescence spectrophotometer. The relative LIP level as compared to WT untreated control was calculated and presented as mean ± SD; n=3 (**, *p* < 0.01 vs. the untreated cells of same genotype; &; *p* < 0.05 vs. the untreated vector controls; ##, *p* < 0.01 vs. the H_2_O_2_-treated vector controls).

The state of MMP is an indicator of the metabolic activity of mitochondria and closely relates to mitochondrial dysfunction and ROS overproduction-induced apoptosis [39]. To examine the underlying protective mechanism of FtMt on mitochondria, the MMP of the three different cell lines was measured. The results showed that H_2_O_2_ treatment decreased MMP by approximately 36% and 38% in SH-SY5Y and vector-SY5Y cells (*P* < 0.05, Fig. 3B), respectively, as compared with the untreated control groups; however, in FtMt-SY5Y cells, the MMP decreased only about 12% (Fig. 3B). These results indicate that FtMt maintains the MMP and protects against the mitochondrial damage induced by H_2_O_2_.

### FtMt significantly decreased intracellular labile iron pool (LIP) levels

Excess ferrous ion (Fe^2+^) can result in the generation of ROS, which damages cellular macromolecules including proteins, lipids and DNA, and consequently triggers apoptosis [6-8, 46]. It has been shown that FtMt could mobilize iron into mitochondria and dramatically redistributes intracellular iron [16, 20]. To further clarify FtMt’s protective mechanism on cell death, the LIP levels in different cell lines were quantified by using iron-specific fluorescence probe calcein-AM. Accessible free iron quenched by calcein was quantified and shown in Fig. 4. The FtMt overexpressing cells indeed had lower amount of LIP as compared with the WT SH-SY5Y cells (*P* < 0.05, indicated by &). H_2_O_2_ treatment dramatically raised the LIP levels in WT and vector transfected SH-SY5Y cells (*P* < 0.01, indicated by **), but it only led to a minor increase in the LIP levels in FtMt overexpressing cells. These results infer that the largely increased LIP in SH-SY5Y cells, following H_2_O_2_ treatment, results in the increased ROS and apoptosis as observed above. The protective role of FtMt overexpression on H_2_O_2_-induced elevation in LIP is remarkably significant.

### FtMt inhibited H_2_O_2_-induced elevations in iron uptake and reductions in iron release

**Figure 5.**
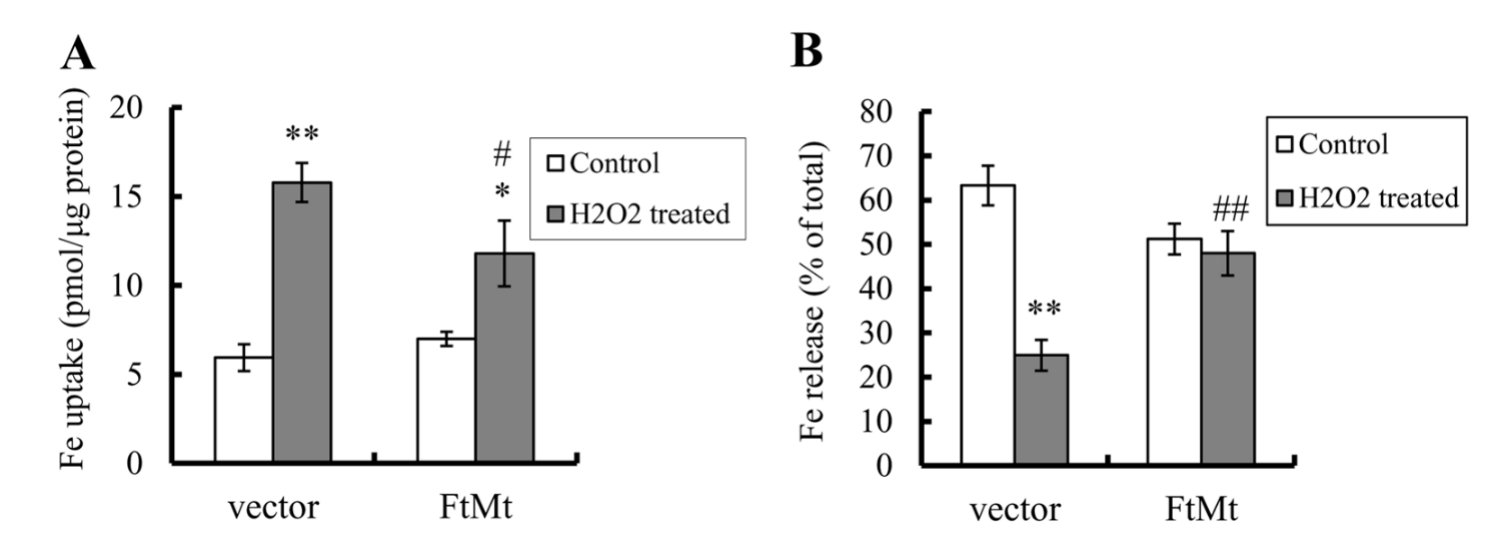
FtMt overexpression attenuated the H_2_O_2_-induced increase of ^55^Fe uptake and decrease of ^55^Fe release Detailed determinations were described in “Materials and methods”. (**A**) ^55^Fe uptake levels were shown as iron concentrations. Data represent means ± SD; n=3 (*, *P* <0.05, and **, *P* < 0.01 vs. the untreated cells of same genotype; #, *p* < 0.05 vs. the H_2_O_2_-treated vector controls). (**B**) ^55^Fe release levels were the percentages of iron in supernatant of cell culture against total iron (sum of supernatant and cell lysate). Data represent means±SD; n=3 (**, *P* < 0.01 vs. the untreated cells of same genotype; ##, *p* < 0.01 vs. the H_2_O_2_-treated vector controls).

Iron-induced oxidative stress can be very destructive because of a positive-feedback loop developed from the release of more free iron from the iron-containing proteins, such as ferritin, heme proteins and Fe-S clusters [7]. As a result, the toxic effect of iron overload in neuronal cells is exacerbated. To clarify the mechanisms of FtMt in regulating intracellular iron homeostasis and the consequent neuroprotective role in neuronal cells, the iron uptake and release were determined by ^55^Fe isotope tracer experiments in vector-SY5Y cells and FtMt-SY5Y cells after treated with H_2_O_2_. The results showed that the cellular ^55^Fe uptake increased significantly in all cell lines after H_2_O_2_ treatment (*P* < 0.01, Fig. 5A), but the increased amount in FtMt-SY5Y cells is much less than that of the vector-SY5Y controls (*P* < 0.05, indicated by ##). The ^55^Fe release in the control vector-SY5Y cells decreased significantly (P<0.01, Fig. 5B), whereas iron release in FtMt-SY5Y cells only slightly decreased. These results suggest that H_2_O_2_ causes cells to uptake more iron, but release less; therefore, iron accumulates in cytosol. However, when FtMt overexpresses, it partially counteracts these effects and maintained a relatively low cellular iron level, protecting cells from damage.

### FtMt maintained iron transport proteins at relative steady levels

**Figure 6.**
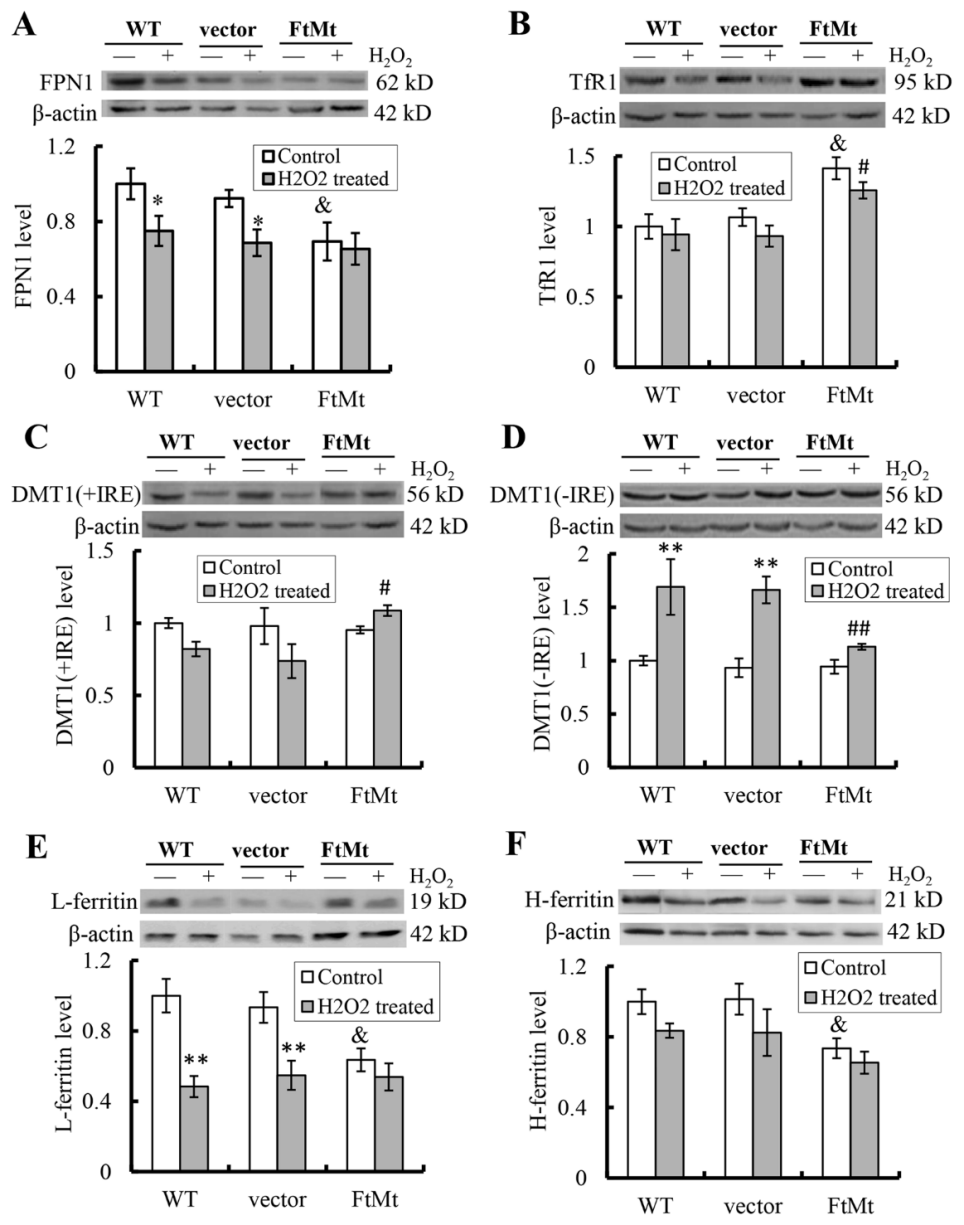
FtMt repressed the alterations of iron release and uptake proteins induced by H_2_O_2_ treatment (100 μM, 24 h) FPN1 (**A**), TfR1 (**B**), DMT1(+IRE) (**C**), DMT1(-IRE) (**D**), L-ferritin (**E**) and H-ferritin (**F**) levels were determined by western blots. A representative blot image for each protein and its respective β-actin was shown. The expression levels in different groups were calculated by normalizing the specific bands to their respective β-actin bands. The relative expression levels as compared to WT untreated control group were calculated and presented as means ± SD, n=6 (*, *p* <0.05 vs. the untreated cells of same genotype; &, *p* < 0.05 vs. the untreated vector controls; #, *p* < 0.05 and ##, *p* < 0.01 vs. the H_2_O_2_-treated vector controls).

To explore the protective function of FtMt on oxidative damage at molecular level, we examined the alterations of iron transport proteins induced by H_2_O_2_ treatment in different cell lines. In SH-SY5Y and vector-SY5Y cells, following H_2_O_2_ treatment, the iron efflux protein ferroportin 1 (FPN1) significantly decreased (*P* < 0.05, Fig. 6A). The iron uptake proteins, transferrin receptor 1 (TfR1) and divalent metal transporter 1 with iron responsive element (DMT1(+IRE)), didn’t show significant changes (Fig. 6B and 6C), but DMT1 without iron responsive element (DMT1(-IRE)) increased dramatically (*P* < 0.01, Fig. 6D). The changes in FPN1 and DMT1(-IRE) may account for the observed intracellular iron accumulation in SH-SY5Y cells caused by H_2_O_2_ treatment [31].

In contrast to SH-SY5Y cells and vector-SY5Y cells, H_2_O_2_ treatment didn’t significantly alter FPN1, TfR1, DMT1(+IRE) and DMT1(-IRE) levels in FtMt-SY5Y cells (Fig. 6A-D). The expression levels of TfR1, DMT1(+IRE) and DMT1(-IRE) in H_2_O_2_ treated FtMt-SY5Y cells were significantly different from that in treated vector-SY5Y cells (indicated by # or ##, *P* < 0.05 or 0.01, respectively, Fig. 6B-D). These results demonstrated that FtMt overexpressing cells maintained the iron transport proteins at relatively steady levels, protecting them from damages induced by H_2_O_2_.

In addition, we found that, prior to H_2_O_2_ treatment, the FtMt-SY5Y cells alone had lower FPN1 expression (*P* < 0.05, indicated by &, Fig. 6A) and higher TfR1 expression (*P* < 0.05) than the untreated vector-SY5Y controls. These findings were consistent with our previous observations [23] and may attribute to cellular regulations in response to low LIP level caused by FtMt overexpression.

### FtMt inhibited H_2_O_2_-induced reductions in cytosolic ferritin levels

The effects of H_2_O_2_ treatment on cytosolic ferritin levels in different cells were also determined. As shown in Fig. 6E, L-ferritin levels in SH-SY5Y and vector-SY5Y cells significantly decreased after H_2_O_2_ treatment (*P* < 0.01). However, the FtMt overexpressing cells had low levels of cytosolic L-ferritin, despite with or without H_2_O_2_ treatment. The H-ferritin levels in all three cells decreased slightly, but not statistically significant (Fig. 6F). These results indicated that H_2_O_2_ induced downregulations of cytosolic ferritins, particularly L-ferritin, and that FtMt overexpression counteracted these effects. The FtMt-SY5Y cells had low levels of cytosolic ferritins, which were consistent with their low intracellular LIP levels, attesting the iron redistributing function of FtMt.

## DISCUSSION

At normal condition, cells were in a balance between ROS and antioxidant. Once the balance was disrupted, cells would be exposed to oxidative stress damage. ROS destroys the enzymes in cytosol, induces lipid peroxidation, decreases MMP, and further leads to cell apoptosis. Iron is one of the important activators that lead to oxidative stress damage [6, 46]. Thus, the iron level in the body is under strict control, and dysregulation of iron homeostasis could cause severe diseases. FtMt, as an iron-storage protein in mitochondria, has been reported to be involved in iron redistribution from cytosol to mitochondria [15, 16, 20]. Recently, FtMt was mainly recognized for its protective role in diseases associated with iron-dependent oxidative damage [23-25, 27-29], although that FtMt sensitized cells to oxidative stress was also reported [47]. Our previous studies have indicated the neuroprotective role of FtMt in Parkinson’s disease and Alzheimer’s disease [23, 27]. In this study, we explored the effects of FtMt on H_2_O_2_ induced neuronal cell damage and further investigated FtMt’s functions at the molecular level.

**Figure 7.**
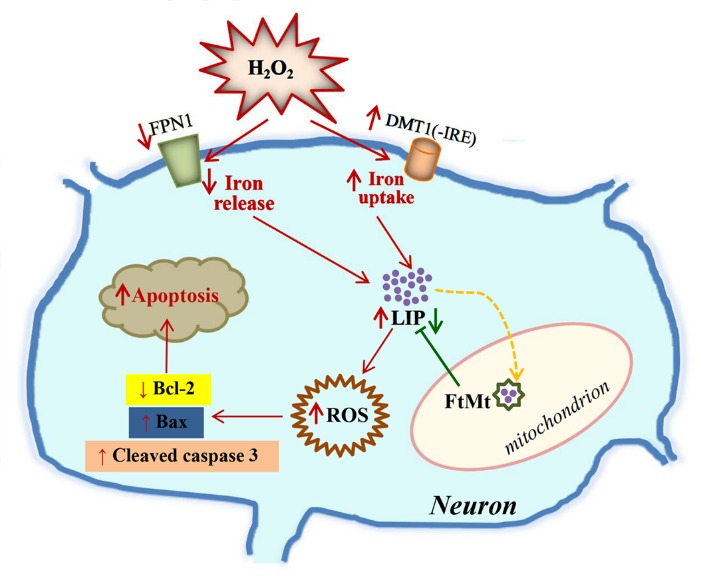
A schematic representation of the proposed neuroprotective mechanism of FtMt on H_2_O_2_-induced neuronal cell damage Extracellular H_2_O_2_ changes the expression of iron-related proteins, particularly iron-release protein FPN1 and iron-uptake protein DMT1(-IRE), resulting in an increase in the intracellular LIP level. The free iron may donate electrons for the generation of ROS, and then cell is triggered to begin the process of programmed cell death, which involves the signalings of Bcl-2, Bax and caspase 3. Alternatively, the overexpressed FtMt may withdraw iron from the cytoplasmic pool, decreasing LIP level. This in turn attenuates H_2_O_2_-induced neurotoxicity and reduces oxidative damage. The damaging effects caused by H_2_O_2_ treatment were indicated with red arrows, while the protective effect of FtMt on LIP level was indicated by a green arrow.

An FtMt overexpressing neuroblastoma SH-SY5Y cell line is used to better investigate the functions of FtMt. Our results indicated that overexpression of FtMt significantly protected neuroblastoma cells from H_2_O_2_ induced apoptosis, which was due to the restriction effects of FtMt on the increase of intracellular ROS and the decrease of MMP, thus protecting cells from oxidative stress-mediated apoptosis. On the molecular level, we found that H_2_O_2_ decreased the expression of Bcl-2 and increased the level of Bax dramatically in SH-SY5Y and vector-SY5Y cells, while the changes of Bcl-2/Bax ratio in FtMt-SY5Y cells were much smaller. Our results also showed that caspase 3, another important member of the apoptotic family, was activated by H_2_O_2_ treatment in SH-SY5Y and vector-SY5Y cells, leading to caspase 3-dependent cell apoptosis. However, in the FtMt overexpressing cells, no obvious increase in cleaved-caspase 3 level was observed.

It has been reported that H_2_O_2_ can modulate cellular iron metabolism [48, 49]. It affects cellular iron acquisition by both IRP1-dependent and -independent mechanisms, and modulates intracellular iron distribution at a time-dependent manner by unknown mechanisms [48]. Consistent with this, our results showed that after H_2_O_2_ treatment, cellular LIP levels greatly increased in SH-SY5Y and vector-SY5Y cells, reaching more than 2-times of the untreated cells. This change may attribute to two aspects. One is the observed higher iron uptake and lower release, correlating with lower expression of iron efflux protein FPN1 and higher iron absorption protein DMT1(-IRE); the other reason might be the internal iron release from various intracellular iron-containing sources, including ferritin, induced by oxidative stress after H_2_O_2_ treatment [47, 48, 50].

The reduction of FPN1 is consistent with the newly published study by Dev, *et al.* [31], in which the role of extracellular H_2_O_2_ in regulation of iron homeostasis-related genes was exhaustively investigated. They concluded that the substantially reduced FPN1 may be responsible for iron accumulation in the H_2_O_2_ treated cells, whereas the alterations of other iron-transport proteins were not obvious. However, they only determined total DMT1 levels, which didn’t alter much. Here, we found that the DMT1(+IRE) showed a statistically insignificant decrease following H_2_O_2_ treatment, which was likely due to the increased iron-binding activity of IRP1 as reported previously [47, 50]. But interestingly, we found that the IRP-independent DMT1(-IRE) expressed to a significantly higher level. DMT1(-IRE) is an important protein involved in iron transport, however, its molecular mechanism was still unknown [51, 52]. We hypothesized that the increased expression of DMT1(-IRE) on cell membrane may account for the increase of iron absorption observed in our ^55^Fe isotope tracer experiments; in addition, the DMT1 on membrane of cell organelles, such as membranes of lysosome and mitochondria [53, 54], may also elevate, likely playing important roles in iron redistribution.

In FtMt-SY5Y cells, we found the LIP levels significantly reduced. However, it seems that the iron acquisition increased and iron release decreased as indicated by the higher TfR1 and lower FPN1 expression. These suggested that the increased iron influx was preferentially transferred into mitochondria and incorporated into FtMt rather than into cytosol [16, 20], which explains the decreased cytosolic LIP even with higher iron acquisition and lower iron release. Despite the differences of FtMt-SY5Y cells from WT SH-SY5Y on iron metabolism, the over-expressed FtMt protected H_2_O_2_-induced elevation in LIP in FtMt cells. Similar to the WT or vector controls, H_2_O_2_ treatment indeed induced higher iron uptake and lower iron release in FtMt-SY5Y cells, however, their LIP level didn’t increase significantly because of the overexpressed FtMt. Consistent with these, both the iron-transport proteins and the cytosolic iron-storage proteins were not altered notably by the extracellular H_2_O_2_ treatment. These all indicated that overexpression of FtMt can cause dramatic redistribution of cellular iron, even at a severe condition, such as extracellular H_2_O_2_ treatment.

In summary, our results showed that FtMt played an important role in rescuing cellular damage induced by H_2_O_2_, which was achieved by regulating iron metabolism (Fig. 7). In SH-SY5Y cells, H_2_O_2_ treatment causes an increase in DMT1(-IRE) expression and decrease in FPN1 expression, resulting in the increased intracellular LIP level. The increased LIP level induces the generation of ROS, which subsequently lead cells to apoptosis, which possibly involves the decrease of Bcl-2/Bax ratio and the activation of apoptosis signal pathway protein caspase 3. In the FtMt overexpressing cells, a great amount of free iron was captured and stored in FtMt, resulting in a substantial decrease in LIP level. Therefore, under H_2_O_2_ treatment, the FtMt overexpressing cells decreased the production of ROS, conserved MMP, maintained the anti-apoptotic protein Bcl-2 level, and inhibited the activation of caspase 3. Consequently, FtMt protected cells from oxidative damage.

Thus, our current study revealed a protective role of FtMt in neuronal cells against H_2_O_2_ induced oxidative damage, which was achieved by modulating the homeostasis of iron metabolism. This observation is consistent with the study by Al-Qenaei *et al.*, in which a defensive effect of FtMt against H_2_O_2_ treatment in Jurkat T cells was implicated [30]. Since dysregulation of iron homeostasis, together with oxidative stress, has been largely demonstrated in several neurodegenerative disorders [3-6, 43], modulation of FtMt expression may prevent or cure these diseases. This process can be greatly facilitated by investigating the mechanisms of regulation of FtMt expression [55, 56]. This study may provide insight into the development of novel effective strategies for treatment and prevention of neurodegenerative diseases caused by iron-dependent oxidative damage.
